# The Effect of Central Venous Catheter Dressings on Preventing Catheter-Related Bloodstream Infections: A Comparative Study of Patients in Intensive Care Post Abdominal Surgery

**DOI:** 10.7759/cureus.113069

**Published:** 2026-07-21

**Authors:** Kursat Dikmen, Enes Aksu, Saygın Altıner, Ozge O Top, Pinar A Yildiz, Huseyin Gobut, Aydın Yavuz, Çagrı Büyükkasap, Hasan Bostanci

**Affiliations:** 1 General Surgery, Gazi University Faculty of Medicine, Ankara, TUR; 2 Infectious Diseases and Clinical Microbiology, Gazi University Faculty of Medicine, Ankara, TUR

**Keywords:** catheter-related bloodstream infection, central venous catheter, chlorhexidine gluconate, dressing, intensive care

## Abstract

Aim: To evaluate the incidence of catheter-related bloodstream infections and the safety characteristics of chlorhexidine gluconate (CHG)-impregnated transparent dressings compared with standard transparent dressings in patients monitored in the intensive care unit post abdominal surgery.

Methods: A total of 102 patients who had a central venous catheter inserted via the internal jugular or subclavian vein and for whom the catheters were expected to remain in place for ≥48 hours were included in the study. Patients with disrupted skin integrity, known allergies to dressing components, or pre-existing bloodstream infections prior to catheterization were excluded. Patients were randomly assigned in a 1:1 ratio to either the CHG-impregnated transparent polyurethane dressing group (n=51) or the standard transparent dressing group (n=51). According to the protocol, dressings were replaced every seven days in the CHG group and every 2-3 days in the standard group, or as clinically necessary. The primary discontinuation was defined as the development of catheter-related bloodstream infections. Categorical variables were analyzed using the chi-square/Fisher test, while independent risk factors were evaluated using logistic regression (p<0.05).

Results: The groups were similar in terms of age, gender, catheterization duration, and intensive care duration. The catheter-related bloodstream infection rate was 5.9% (3/51) in the CHG group and 25.5% (13/51) in the standard group, and the difference was statistically significant (p=0.014); the absolute risk reduction was 19.6%. Multivariate analysis demonstrated that standard dressing use was an independent and strong risk factor for catheter-related bloodstream infection (OR: 21.2; 95% CI: 2.5-177.1; p=0.005). Each additional day of catheterization increased the risk of infection (OR: 1.2 (1.1-1.3); p=0.002 ), and female gender showed an independent association (OR: 8.1 (1.5-44.6); p=0.016). Skin reactions were found in 5.9% (3/51) of the CHG group and 27.5% (14/51) of the standard dressing group, with a significant difference between the groups (p=0.037). Reaction severity was found to be significantly lower in the CHG group (p=0.045). Major complications and dressing intolerance were observed only in the standard dressing group (3.9%), while no such complications were observed in the CHG group (p=0.010).

Conclusion: The routine inclusion of CHG-impregnated transparent dressings in central venous catheter care protocols in intensive care units is considered a powerful strategy that can contribute to reducing the incidence of catheter-related bloodstream infections, improving patient safety, and managing care processes more effectively.

## Introduction

Central venous catheters are widely used in intensive care units to meet vital clinical needs such as hemodynamic monitoring, complex drug therapies, blood and blood product support, and total parenteral nutrition [1]. However, the use of these devices is associated with serious morbidity and mortality complications, primarily catheter-related bloodstream infections (CRBSIs) [1,2]. The development of CRBSI not only negatively impacts patient safety but can also increase mortality rates by up to 35%, prolong hospital stays, and significantly increase the economic burden on the healthcare system [3,4].

Although various mechanisms for catheter contamination have been identified, the most common route is the migration of microorganisms present in the skin flora from the catheter insertion site to the catheter tip [1]. Therefore, ensuring effective skin antisepsis at the catheter insertion site and selecting appropriate dressing methods that prevent microorganisms from entering the subcutaneous catheter tract are fundamental strategies for reducing the risk of infection [5]. Traditionally used sterile gauze and standard polyurethane dressings form a physical barrier, but because they do not possess active antimicrobial properties, they may not always demonstrate sufficient efficacy in infection control [1].

Chlorhexidine gluconate (CHG) is an agent with a broad-spectrum antiseptic effect against the vast majority of pathogens that are the most common cause of bloodstream infections [1,6]. CHG-impregnated dressings have been developed to suppress microbial growth by providing continuous antiseptic release at the catheter insertion site and to minimize the risk of contamination by extending dressing replacement intervals [1]. Current meta-analyses and clinical studies show that CHG-impregnated dressings significantly reduce both catheter tip colonization and the incidence of CRBSIs compared to standard dressings [4,7]. This study aims to evaluate the effects and safety characteristics of CHG-impregnated dressings versus standard dressings on catheter-related infections in a homogeneous group of patients monitored in the intensive care unit post abdominal surgery.

The primary objective of this study was to compare the incidence of CRBSIs in patients in the intensive care unit post abdominal surgery who receive CHG-impregnated transparent dressings versus standard transparent dressings following central venous catheterization. We hypothesized that CHG-impregnated dressings would reduce the incidence of CRBSIs compared with standard transparent dressings. The primary outcome was the incidence of CRBSI. Secondary outcomes included dressing-related safety parameters, including local skin reactions, dressing intolerance, dressing integrity, and other dressing-related complications.

## Materials and methods

Patient allocation

A total of 102 eligible patients were allocated in a 1:1 ratio to either the CHG-impregnated transparent polyurethane dressing group (Difa IV CHG Securement Dressing®, Istanbul, Turkey; n=51) or the standard transparent polyurethane dressing group (n=51). Because the two dressings were visually distinguishable, blinding of patients and healthcare providers was not feasible; therefore, the study was conducted using an open-label design.

The study was conducted in accordance with the ethical principles of the Declaration of Helsinki.

Ethical approval

The study was approved by the Gazi University Ethics Committee (Approval No. 2025-1829, approval date: 21 October 2025).

Catheterization 

All central venous catheterizations were performed by experienced clinicians under maximal sterile barrier precautions, including the use of sterile gloves, sterile gowns, surgical masks, caps, and large sterile drapes. Skin antisepsis was performed using an alcohol-based chlorhexidine solution before catheter insertion. Catheters were secured using silk sutures according to institutional practice.

Dressing protocol

Patients allocated to the intervention group received CHG-impregnated transparent polyurethane dressings, whereas patients in the control group received standard transparent polyurethane dressings without antimicrobial properties. Dressing replacement intervals were determined according to the manufacturers' recommendations and institutional catheter care protocols reflecting routine clinical practice. Accordingly, CHG dressings were routinely replaced every seven days, whereas standard transparent dressings were replaced every 2-3 days or earlier if they became loose, wet, visibly soiled, or clinically indicated.

Outcome measures

The primary outcome of the study was the incidence of CRBSI. CRBSI was diagnosed according to predefined microbiological and clinical criteria, including isolation of the same microorganism from both peripheral blood cultures and catheter tip cultures, compatible clinical findings (fever >38°C and/or hypotension), and the absence of another identifiable source of infection.

Secondary outcomes included dressing-related safety outcomes, including local skin reactions, contact dermatitis, dressing intolerance, dressing integrity, and dressing-related complications. Skin reactions were assessed at each dressing change using a standardized four-point severity scale (0=no reaction, 1=mild, 2=moderate, 3=severe). Major complications were defined as reactions requiring dressing discontinuation or additional medical treatment, whereas transient mild reactions requiring no intervention were classified as minor complications.

Definitions and outcome criteria

The primary endpoint of the study is the incidence of CRBSIs developing after catheterization. A CRBSI is diagnosed based on the following criteria: growth of the same microorganism in a blood culture obtained from a peripheral vein and in a culture obtained from the catheter tip (using quantitative or semi-quantitative methods), the presence of clinical signs of infection in the patient such as fever (>38°C) and/or hypotension, and the absence of any other identifiable source of infection [1].

In the case of secondary application, safety and the tolerability of the dressing were evaluated. The safety assessment was conducted by considering skin reactions developing at the dressing application site, allergic/irritant skin findings, and adverse events affecting the continued use of the dressing.

The presence of erythema, edema, maceration, itching, and pain at the catheter insertion site was systematically recorded at each dressing change. Reaction severity was graded using a standard four-point scale (0: none, 1: mild, 2: moderate, 3: severe) and defined as a skin reaction at the application site [8].

Findings consistent with clinically irritant or allergic contact dermatitis (widespread erythema, vesiculation, marked itching/pain, lesions extending beyond the dressing borders, etc.) were considered contact dermatitis. In some patients, discontinuation of the product and transition to an alternative dressing were planned and recorded as discontinuation. The relationship between adverse events and the dressing was evaluated. Early discontinuation of the dressing due to dressing intolerance (severe pain, widespread reaction, clinically significant dermatitis, or the patient’s inability to tolerate the dressing) was defined as product discontinuation, and rates were compared across groups. Safety features were classified as major or minor based on their clinical significance. Reactions leading to product discontinuation, additional treatment requirements, or clinical intervention were considered major complications (e.g., dermatitis/allergic reaction resulting in product discontinuation), while mild and transient findings not requiring intervention were considered minor complications (e.g., short-term mild erythema).

Statistical analysis

Statistical analyses were performed using IBM SPSS Statistics for Windows, version 25.0 (IBM Corp., Armonk, NY, USA). Continuous variables were assessed for normality before analysis and are presented as either mean ± standard deviation or median with interquartile range (IQR), as appropriate. Categorical variables were compared using the chi-square test or Fisher's exact test. Logistic regression analysis was performed as an exploratory post hoc analysis to identify factors independently associated with CRBSI. Odds ratios (ORs) with 95% confidence intervals (CIs) were calculated, and a two-sided p-value <0.05 was considered statistically significant.

## Results

The demographic characteristics, catheter application characteristics, and clinical outcomes of patients using chlorhexidine-impregnated transparent dressings and standard transparent dressings were compared. The groups showed similar characteristics in terms of age, gender, catheterization duration, and length of stay in the intensive care unit. The CRBSI rate was 5.9% (n:3) in the CHG group and 25.5% (n:13) in the standard dressing group, and the difference was statistically significant (p=0.014). The absolute risk reduction was calculated as 19.6%. However, no significant difference was observed between the groups in terms of mortality rates. The catheters were inserted by a radiologist, an anesthesiologist, and a surgeon (Table 1).

**Table 1:** Comparison of demographic and clinical characteristics of patients with CHG-impregnated dressings and standard dressings The numerals under the column entries "Catheterization by" and "Catheter site" represent the following: 1 = radiologist, 2 = anesthesiologist, 3 = surgeon. CHG, chlorhexidine gluconate; N/A, not applicable.

Variable	CHG (n=51)	Normal (n=51)	p-Value
Age (years), median (IQR)	66.0 (56.0-71.0)	65.0 (51.0-75.0)	0.923
Sex, n (%)			
Female	25 (49.0%)	30 (58.8%)	0.427
Male	26 (51.0%)	21 (41.2%)	
Comorbid disease, n (%)			
No	6 (11.8%)	14 (27.5%)	0.081
Yes	45 (88.2%)	37 (2.5%)	
Catheterization by, n (%)			
1	6 (11.8%)	11 (21.6%)	0.056
2	35 (68.6%)	23 (45.1%)	
3	10 (19.6%)	17 (33.3%)	
Catheter site, n (%)			
1	44 (86.3%)	46 (90.2%)	N/A
2	1 (2.0%)	0 (0.0%)	
3	6 (11.8%)	5 (9.8%)	
Mechanical ventilator, n (%)			
No	44 (86.3%)	40 (78.4%)	0.436
Yes	7 (13.7%)	11 (21.6%)	
Catheter infection, n (%)			
No	48 (94.1%)	38 (74.5%)	0.014
Yes	3 (5.9%)	13 (25.5%)	
Mortality, n (%)			
No	40 (78.4%)	34 (66.7%)	0.267
Yes	11 (21.6%)	17 (33.3%)	
Catheterization time (days), median (IQR)	8.0 (6.0-15.0)	7.0 (5.0-12.0)	0.370
ICU stay, median (IQR)	2.0 (2.0-5.0)	3.0 (2.0-6.0)	0.442

When comparing the clinical characteristics of patients who developed CRBSIs with those who did not, it was found that the catheter duration and intensive care unit stay duration were significantly longer in patients who developed CRBSIs (p=0.020 and p=0.002, respectively). Furthermore, a significant relationship was determined between the development of infection and the type of dressing, and the rate of CRBSIs was found to be higher in patients with standard dressings (p=0.014). It was determined that female gender was associated with the development of CRBSIs (p=0.034) (Table 2).

**Table 2:** Comparison of demographic and clinical characteristics of patients who developed catheter-related infections and those who did not The numerals under the column entries "Catheterization by" and "Catheter site" represent the following: 1 = radiologist, 2 = anesthesiologist, 3 = surgeon. CHG, chlorhexidine gluconate; N/A, not applicable.

Variable	No catheter-related infection (n= 86)	Catheter-related infection (n=16)	p-Value
Age (years), median (IQR)	65.0 (51.8-71.0)	68.5 (59.3-75.0)	0.277
Sex, n (%)			
Female	42 (76.4%)	13 (25.6%)	0.034
Male	44 (93.6%)	3 (6.4%)	
Comorbid disease, n (%)			
No	18 (90.0%)	2 (10.0%)	0.732
Yes	68 (79.1%)	14 (87.5%)	
Catheterization by, n (%)			
1	12 (70.6%)	5 (29.4%)	0.147
2	52 (89.7%)	6 (10.3%)	
3	22 (81.5%)	5 (18.5%)	
Catheter site, n (%)			
1	77 (85.6%)	13 (14.4%)	N/A
2	0 (0.0%)	1 (100.0%)	
3	9 (81.8%)	2 (18.2%)	
Dressing type			
CHG	48 (94.1%)	3 (5.9%)	0.014
Normal	38 (74.5%)	13 (25.5%)	
Mechanical ventilator, n (%)			
No	73 (86.9%)	11 (13.1%)	0.152
Yes	13 (72.2%)	5 (27.8%)	
Catheterization time (days), median (IQR)	7.0 (5.0-12.3)	13.0 (6.0-24.0)	0.020
ICU stay, median (IQR)	2.0 (2.0-5.0)	5.0 (3.0-9.8)	0.002

Independent risk factors associated with the development of CRBSIs are shown in Table 3. Logistic regression analysis demonstrated that standard dressing use is a strong independent risk factor for the development of CRBSIs (OR: 21.2; 95% CI: 2.5-177.1; p=0.005). Each additional day of catheterization increased the risk of infection (OR: 1.2; p=0.002), and female gender was also identified as an independent factor associated with the development of infection (OR: 8.1; p=0.016).

**Table 3:** Logistic regression analysis of independent risk factors associated with the development of catheter-related infections CHG, chlorhexidine gluconate.

Variable	OR (95% CI)	p-Value
Sex		
Female	8.1 (1.5-44.6)	0.016
Male	Ref	
Catheterization time	1.2 (1.1-1.3)	0.002
Dressing type		
CHG	Ref	0.005
Normal	21.2 (2.5-177.1)	

The incidence of skin reactions was 5.9% (n:3) in the CHG group and 27.5% (n:14) in the standard dressing group, with a significant difference between the groups (p=0.037). Not all patients developed skin reactions, when the severity scores of the 17 patients who developed skin reactions were evaluated, reaction severity was found to be significantly lower in the CHG group (p=0.045). Major complications and dressing intolerance were observed only in the standard dressing group (3.9%, n:2), while no such complications were observed in the CHG group (p=0.010). The incidence of dressing integrity compromise was 3.9% (n:2) in the CHG group and 13.7% (n:7) in the standard dressing group, and the difference was statistically significant (p=0.023) (Table 4).

**Table 4:** Comparison of safety and dressing-related complication characteristics CHG, chlorhexidine gluconate.

Complication	CHG dressing (n=51)	Standard dressing (n=51)	p-Value
Skin reaction, n (%)			
Yes	3 (5.9%)	14 (27.5%)	0.037
No	48 (94.1%)	37 (2.5%)	
Skin reaction score, n (%)			
Mild	2 (3.9%)	8 (15.7%)	0.045
Moderate	1 (2.0%)	4 (7.8%)	
Severe	-	2 (3.9%)	
Major complication, n (%)			
Yes	-	2 (3.9%)	0.010
No	51 (100%)	49 (96.1%)	
Dressing intolerance, n (%)			
Yes	-	2 (3.9%)	0.010
No	51 (100%)	49 (96.1%)	
Disruption of dressing integrity, n (%)			
Yes	2 (3.9%)	7 (13.7%)	0.023
No	49 (96.1%)	44 (86.3%)	

## Discussion

This study demonstrates that CHG-impregnated transparent dressings used in abdominal surgery patients monitored in the intensive care unit significantly reduce the risk of CRBSIs compared to standard transparent dressings [1,4,5]. According to the clinical data obtained, the use of standard transparent dressings increases the risk of infection by approximately 21.2 times compared to CHG-impregnated dressings. This finding is consistent with systematic reviews in the Cochrane database and the results of large-scale meta-analyses reporting that drug-impregnated dressings reduce the incidence of CRBSIs [2,6]. In particular, a study reported from Turkey [3] and a recent meta-analysis by Xu et al. [4] reported that CHG-impregnated dressings significantly reduce both the incidence of CRBSIs and the risk of catheter tip colonization compared to standard polyurethane dressings.

The primary mechanism of catheter contamination is the migration of microorganisms (particularly *Staphylococcus epidermidis* and *Staphylococcus aureus*) from the skin flora toward the catheter tip from the catheter insertion site [1,7,9]. CHG is a cationic biguanide with broad-spectrum antiseptic activity against most causative agents of CRBSIs [8,10]. In our study, the infection rate in the CHG group remained at a low level of 5.9%, which can be explained by chlorhexidine's continuous suppression of microbial proliferation in the skin flora. In a similar study reported by Ergul et al., the use of CHG-impregnated dressings was shown to reduce infections caused by Gram-positive microorganisms from 8.8% to 0%. These findings support the critical importance of the chlorhexidine barrier in a patient group with compromised skin integrity after surgery and susceptible to skin flora contamination [3]. Similar reductions in CRBSIs with chlorhexidine-containing dressings have also been demonstrated in randomized clinical trials and meta-analyses in critically ill patients [11,12].

Another important finding of our study is the strong relationship between the duration of catheterization and the development of infection. It is known in the literature that prolongation of catheterization time significantly increases the risk of infection [13,14]. CHG-impregnated dressings can continuously release the active substance for up to seven days, allowing for the safe extension of dressing replacement intervals [15]. Indeed, Timsit et al. demonstrated in a randomized controlled trial that CHG-impregnated sponges, when used with less frequent dressing replacements, reduced the risk of catheter-related sepsis in critically ill patients. Additionally, securing the catheter with silk sutures or new-generation fixation methods supports this protective effect by reducing mechanical movement [8].

This study demonstrated that CHG-impregnated transparent dressings not only reduce the incidence of CRBSIs but also offer a superior profile in terms of safety and tolerability compared to standard transparent dressings. Findings related to local skin reactions, dressing intolerance, and dressing integrity complications provide important safety data supporting the integration of CHG dressings into intensive care practice.

Application-site skin reactions were detected at a low rate of 5.9% in the CHG group, while this rate reached 27.5% in the standard dressing group, and the difference was found to be statistically significant (p=0.037). Reaction severity was also found to be significantly lower in the CHG group (p=0.045). The occurrence of moderate to severe reactions in the standard dressing group demonstrates the effect of dressing material biocompatibility on clinical outcomes, particularly in patient populations with increased post-surgical inflammatory response.

Major complications and dressing intolerance were observed only in the standard dressing group (3.9%), while no such complications were observed in the CHG group (p=0.001). This demonstrates that CHG-impregnated dressings better preserve patient comfort and significantly reduce the risk of adverse events that could compromise dressing continuity. In clinical practice, early discontinuation of the product due to dressing intolerance may lead to increased dressing replacement frequency, resulting in more frequent manipulation of the catheter insertion site and consequently a secondary increase in the risk of infection. In this context, the absence of product discontinuation requirements in the CHG group highlights the operational superiority of these dressings.

The rate of dressing integrity disruption was 3.9% in the CHG group and 13.7% in the standard dressing group, and this difference was found to be statistically significant (p=0.023). Maintaining the integrity of the dressing acts as an indirect barrier to prevent infection by limiting exposure of the catheter insertion site to environmental contamination. This finding demonstrates that CHG dressings provide clinical benefits not only in terms of antimicrobial activity but also in terms of mechanical stability.

Studies reported by Timsit et al. [8] and Margatho et al. [5] in the literature show that CHG-impregnated dressings are associated with lower rates of local complications and better dressing integrity. The findings of this study strongly confirm these advantages in patients in intensive care post abdominal surgery in terms of both infection prevention and safety/tolerability.

The strengths of this study include its prospective comparative design, the evaluation of two dressing strategies used in routine clinical practice, the use of predefined microbiological criteria for the diagnosis of CRBSIs, and the simultaneous assessment of clinically relevant safety outcomes, including skin reactions, dressing intolerance, and dressing integrity.

This study has several limitations. First, it was conducted at a single tertiary-care center with a relatively small sample size, which may limit the generalizability of the findings. Second, because the CHG-impregnated and standard transparent dressings were visually distinguishable, blinding was not feasible, introducing the potential for observer bias, particularly for subjective safety outcomes. Although the primary outcome was based on predefined microbiological criteria, some degree of assessment bias cannot be completely excluded. Third, dressing replacement intervals differed between the two groups in accordance with routine clinical practice, resulting in less frequent catheter manipulation in the CHG group. Therefore, it is difficult to determine the extent to which the observed reduction in CRBSIs was attributable to the antimicrobial properties of chlorhexidine alone, as reduced catheter manipulation may also have contributed to the observed benefit. Finally, because the study included only patients in intensive care post abdominal surgery, the findings should be interpreted with caution when applied to other patient populations. Larger multicenter randomized studies are warranted to confirm these findings.

## Conclusions

In conclusion, CHG-impregnated transparent dressings were associated with a significantly lower incidence of CRBSIs compared to standard transparent dressings in patients monitored in the intensive care unit after abdominal surgery. In addition to their effectiveness in infection prevention, CHG dressings were associated with fewer dressing-related adverse events in our study. However, these findings should be interpreted with caution because of the limited number of observed safety events and previous reports describing CHG-associated contact dermatitis.

These findings suggest that the use of CHG-impregnated transparent dressings may represent a beneficial strategy in central venous catheter care. Incorporating these dressings into routine intensive care unit catheter management protocols may contribute to reducing CRBSIs in selected patient populations. Further adequately powered multicenter studies are required to confirm these findings and better define the safety profile of CHG-impregnated dressings.
